# Investigation on the Protective Effects of Cranberry Against the DNA Damage Induced by Benzo[a]pyrene

**DOI:** 10.3390/molecules17044435

**Published:** 2012-04-12

**Authors:** Eduardo Madrigal-Santillán, Sonia Fragoso-Antonio, Carmen Valadez-Vega, Gloria Solano-Solano, Clara Zúñiga Pérez, Manuel Sánchez-Gutiérrez, Jeannett A. Izquierdo-Vega, José Gutiérrez-Salinas, Jaime Esquivel-Soto, César Esquivel-Chirino, Teresa Sumaya-Martínez, Tomas Fregoso-Aguilar, Jorge Mendoza-Pérez, José A. Morales-González

**Affiliations:** 1 Institute of Health Sciences, Autonomous University of Hidalgo State, Ex-Hacienda de la Concepción. Pachuca, Hidalgo, 42080, Mexico; Email: cachito_sony@yahoo.com.mx (S.F.-A.); m.valadezvega@lycos.com (C.V.-V.); glorias_4020@yahoo.com.mx (G.S.-S.); zupecl@yahoo.com.mx (C.Z.P.); spmtz68@yahoo.com.mx (M.S.-G.); jizquierdovega@gmail.com (J.A.I.-V.); jmorales101@yahoo.com.mx (J.A.M.-G.); 2 Laboratory of Biochemistry and Experimental Medicine, Division of Biomedical Research, National Medical Center “20 de Noviembre”, ISSSTE. México D.F., 03229, Mexico;Email: quauhtlicutli@yahoo.com; 3 Faculty of Dentistry, School Circuit S/N. UNAM. México D.F., 04510, Mexico; Email: jaime_esquivel2003@hotmail.com (J.E.-S.); cesquivelch@gmail.com (C.E.-C.); 4 Secretary of Research and Graduate Studies, Autonomous University of Nayarit, “City of the culture Amado Nervo”, Boulevard Tepic-Xalisco S/N. Tepic, Nayarit, 28000, Mexico; Email: teresumaya@hotmail.com; 5 National School of Biological Sciences, IPN. Av. Wilfrido Massieu. Unidad A. López Mateos. Zacatenco. México D.F., 07700, Mexico; Email: fisiobiologo@hotmail.com (T.F.-A.); jorgemendozaperez@yahoo.com (J.M.-P.)

**Keywords:** cranberries, benzo[a]pyrene, micronucleus assay, antigenotoxic effect

## Abstract

There are few reports that demonstrate the antigenotoxic potential of cranberries. Although the types of berry fruits consumed worldwide are many, this paper focuses on cranberries that are commonly consumed in Mexico (*Vaccinium macrocarpon* species). The purpose of the present study is to determine whether cranberry ethanolic extract (CEE) can prevent the DNA damage produced by benzo[a]pyrene (B[a]P) using an *in vivo* mouse peripheral blood micronucleus assay. The experimental groups were organized as follows: a negative control group (without treatment), a positive group treated with B[a]P (200 mg/kg), a group administered with 800 mg/kg of CEE, and three groups treated with B[a]P and CEE (200, 400, and 800 mg/kg) respectively. The CEE and benzo[a]pyrene were administered orally for a week, on a daily basis. During this period the body weight, the feed intake, and the determination of antigenotoxic potential were quantified. At the end of this period, we continued with the same determinations for one week more (recovery period) but anymore administration of the substances. The animals treated with B[a]P showed a weight increase after the first week of administration. The same phenomenon was observed in the lots combined with B[a]P and CEE (low and medium doses). The dose of 800 mg/kg of CEE showed similar values to the control group at the end of the treatment period. In the second part of the assay, when the substances were not administered, these experimental groups regained their normal weight. The dose of CEE (800 mg/kg) was not genotoxic nor cytotoxic. On the contrary, the B[a]P increases the frequency of micronucleated normochromatic erythrocytes (MNNE) and reduces the rate of polychromatic erythrocytes (PE) at the end of the treatment period. With respect to the combined lots, a significant decrease in the MN rate was observed from the sixth to the eighth day of treatment with the two high doses applied; the highest protection (60%) was obtained with 800 mg/kg of CEE. The same dose showed an anticytotoxic effect which corresponded to an improvement of 62.5% in relation to the animals administered with the B[a]P. In the second period, all groups reached values that have been seen in the control group animals. Our results suggest that the inhibition of clastogenicity of the cranberry ethanolic extract against B[a]P is related to the antioxidant capacity of the combination of phytochemicals present in its chemical composition.

## 1. Introduction

Diverse plants and fruits have been used effectively throughout history to treat a variety of illnesses. This practice has led to the synthesis of specific compounds with therapeutic properties [1]. The interest in such a practice has recently strengthened due to the knowledge that chemicals such as proteases and antioxidants may prevent or reduce the development of cancer by blocking genes, like the *ras* oncogene [2]. Among small soft-fleshed colorful fruits, berries make up the largest proportion consumed in our diet. Berry fruits are popularly consumed not only in fresh and frozen forms, but also as processed and derived products including canned fruits, yogurts, beverages, jams, and jellies. In addition, there has been a growing trend in the intake of berry extracts as ingredients in functional foods and dietary supplements, which may or may not be combined with other colorful fruits, vegetables, and herbal extracts [3]. Berry fruits commonly consumed in America include blackberries (*Rubus spp*.), black raspberries (*Rubus occidentalis*), red raspberries (*Rubus idaeus*) and strawberries (*Fragaria x ananassa*), blueberries (*Vaccinium corymbosum*), and cranberries (*Vaccinium macrocarpon*). Other “niche-cultivated” berries and forest/wild berries, for example, bilberries, black currant, lingonberry, and cloudberry, are also popularly consumed in other regions of the World [3,4]. The North American cranberry (*Vaccinium macrocarpon*) is of a growing public interest as a functional food because of potential health benefits linked to phytochemicals of the fruit. Cranberry juice has long been consumed for the prevention of urinary tract infections, and research linked this property to the ability of cranberry proanthocyanidins to inhibit the adhesion of *Escherichia coli* bacteria responsible for these infections [5,6]. These studies, which brought to light the unique structural features of cranberry proanthocyanidins [7], have sparked numerous clinical studies probing a cranberry´s role in the prevention of urinary tract infections and targeted the nature of the active metabolites. Further antibacterial adhesion studies demonstrated that cranberry constituents also inhibit the adhesion of *Helicobacter pylori*, a major cause of gastric cancer, to human gastric mucus [8]. The earliest report of potential anti-carcinogenic activity appeared in 1996 in the University of Illinois [9]. Extracts of cranberry and bilberry were observed to inhibit ornithine decarboxylase (ODC) expression and induce the xenobiotic detoxification enzyme quinone reductase *in vitro* [9]. Subsequent studies with cranberry and other berries in cellular models have focused on some cancers such as breast, colon, liver, prostate and lung [10,11,12,13,14,15]. This biological activity of berries are partially attributed to their high content of a diverse range of phytochemicals such as flavonoids (anthocyanins, flavonols, and flavanols), tannins (proanthocyanidins, ellagitannins, and gallotannins), quercetin, phenolic acids, lignans, and stilbenoids (e.g., resveratrol) [10]. With respect to this genotoxic and/or antigenotoxic potential, there are few reports in the literature that demonstrate this effect and the majority of studies were performed *in vitro* cell culture models [16,17,18,19,20,21]. Boateng *et al*. demonstrated that consumption of some juices of berries (as blueberries, blackberries, and cranberry) can reduce the formation of aberrant crypt foci (ACF) induced by azoxymethane in Fisher male rats [22]. Another study, in which it was administrated a lyophilized extract of *Vaccinium ashei* berries in male Swiss mice during 30 days, showed to have improved the performance on memory tasks and has a protective effect on the DNA damage in brain tissue evaluated with the comet assay [23]. There are some precedents that quercetin (a natural flavonoid commonly detected in cranberries, and blueberries) is not a genotoxic compound; on the contrary, it reduces significantly the oxidative damage to DNA induced by the exposure of H_2_O_2_ evaluated in Caco-2 and Hep G2 cells [24,25]. Although the types of berry fruits consumed worldwide are many, this paper focuses on cranberries that are commonly consumed in Mexico, especially in the states of Tlaxcala, Hidalgo, and Puebla. The purpose of the present study is to determine whether cranberry ethanolic extract can prevent the DNA damage produced by benzo[a]pyrene using an *in vivo* mouse peripheral blood micronucleus assay.

## 2. Results

Table 1 shows the results obtained for the weight of the animals. A weight increase was observed in the control animals starting from the second day and maintained throughout the subsequent days. An increase of 6.5 g was observed at the end of this experimental period. During the second (recovery) period, the same tendency was observed with a final mean weight of 36.13 g. A similar behavior was found with respect to the animals treated with CEE, suggesting that this chemical produced no toxicity. Moreover, the animals treated with B[a]P showed a statistically significant weight increase at the end of the first period (a mean of 2.0 g more than the weight with respect to the control group was found in the eighth day). During the recovery phase, these animals reached a weight similar to the control group. Finally, the animals treated with the combination of compounds (CEE plus benzo[a]pyrene) showed a different response: the low and medium dose of CEE showed a similar effect to the B[a]P group (however, any of these doses were statistically significant from other group), whereas the dose of 800 mg/kg of CEE produced a protective effect at the end of the treatment period. In the second part of the assay, when the substances were not administered anymore, these experimental groups regained their normal weight.

The quantity of food consumed by mice used in the experiment is shown in Table 2. The control mice as well as those administered with CEE had similar food consumption throughout the experiment. On the contrary, the benzo[a]pyrene-treated mice showed a tendency to consume more food than the control animals at the end of the first period and they reduced the consumption during the second period. The same effect was observed in the animals treated with CEE (200 mg/kg) plus benzo[a]pyrene. The other two groups treated with both chemicals (CEE and B[a]P) showed a food intake similar to the control group. During the recovery period all groups consumed similar amounts of food.

The frequency of MNNE in the studied groups is shown in Table 3. Animals belonging to the control and CEE groups had no MN increase in the 14 days of the experiment; the mean value was 0.7 MN/1,000 NE. The result suggests that the cranberry ethanolic extract is not genotoxic. On the other hand, the mice treated with benzo[a]pyrene (200 mg/kg) manifested a significant increase since the fourth day of the assay, with the highest genotoxic damage of 4.8 MN/1,000 NE at the eighth day. Once the mutagen was not administered, a decrease in the rate of MN was observed, reaching a recovery of 80% at the end of the experiment. With respect to the effect of the cranberry on the MN formed by B[a]P, no protection was detected with the low dose tested (200 mg/kg). However, a significant decrease in the MN rate was determined from the sixth to the eighth day of treatment with the two high doses (400 and 800 mg/kg), but the highest protection (approximately, 65%) was obtained with 800 mg/kg of CEE at the eighth day. During the second period, the three groups of animals treated with the combination of CEE plus B[a]P showed a constant MN decrease reaching a recovery value of 100% in comparison to the control level.

Table 4 shows the results obtained from the relation between PE and the number of normochromatic erythrocytes (PE/NE índex). At the beginning of the experiment, the PE/NE índex was similar in all groups; in the 2 successive weeks, the control group as well as the animals treated with 800 mg/kg of CEE showed no significant variation in the index. On the other hand, the mice administered with B[a]P showed a significant reduction in the rate of PE during the last three days of the treatment period (70% with respect to the control group level). The result is similar to that observed in animals treated with the lowest doses of cranberry (200 and 400 mg/kg) plus the mutagen. However, the higher dose of cranberries (800 mg/kg) produced an anticytotoxic effect that corresponded to a recovery of PE/NE index in approximately 62.5% in relation to the animals administered with B[a]P. In the second period, all groups reached values that were seen in the control animals.

**Table 1:** Weight gain (g) in mice treated with cranberry ethanolic extract (CEE) and benzo[a]pyrene (X ± SD).

Day	Control	CEE 800 mg/kg	B[a]P 200 mg/kg	CEE + B[a]P 200 + 200 mg/kg	CEE + B[a]P 400 + 200 mg/kg	CEE + B[a]P 800 + 200 mg/kg
Treatment period						
0	23.39 ± 2.59	23.59 ± 1.29	23.29 ± 0.68	23.12 ± 0.83	23.62 ± 0.68	23.32 ± 1.50
1	25.55 ± 2.32	24.65 ± 1.93	24.75 ± 1.03	24.05 ± 0.72	24.05 ± 1.35	24.75 ± 0.84
2	26.05 ± 2.46	26.80 ± 1.79	25.66 ± 0.99	26.02 ± 0.71	25.99 ± 1.43	25.26 ± 0.82
3	26.79 ± 1.71	26.29 ± 1.99	26.66 ± 1.10	26.71 ± 0.86	26.29 ± 0.19	26.29 ± 0.38
4	27.02 ± 2.02	27.52 ± 1.94	27.02 ± 0.96	27.33 ± 0.87	27.41 ± 1.34	27.13 ± 0.63
5	27.14 ± 2.22	27.84 ± 1.96	27.91 ± 0.92	28.21 ± 1.33	27.91 ± 0.15	27.13 ± 0.63
6	28.69 ± 2.57	28.41 ± 1.80	29.70 ± 0.83	29.21 ± 1.07	29.21 ± 1.47	29.11 ± 0.16
7	29.21 ± 2.50	29.51 ± 1.84	30.83 ± 1.40	30.59 ± 0.37	29.33 ± 1.39	29.98 ± 0.20
8	29.98 ± 2.48	29.51 ± 1.88	31.99 ± 1.33 ^a^	31.23 ± 1.69	31.23 ± 1.11	30.02 ± 0.20
Recovery period						
9	30.59 ± 2.82	30.78 ± 2.13	32.79 ± 1.51 ^a^	32.59 ± 1.54 ^a^	31.23 ± 1.11	30.83 ± 0.10 ^b^
10	31.69 ± 3.02	31.89 ± 2.83	33.13 ± 0.60 ^a^	32.79 ± 0.86	33.03 ± 1.25	31.89 ± 1.18
11	32.89 ± 3.10	32.33 ± 2.83	33.23 ± 1.26	33.63 ± 0.16	33.11 ± 1.15	32.40 ± 0.11
12	34.93 ± 3.10	34.79 ± 2.83	35.20 ± 0.26	35.02 ± 0.36	34.12 ± 1.05	34.50 ± 0.74
13	34.93 ± 3.14	35.20 ± 2.53	35.12 ± 1.16	35.50 ± 0.60	35.20 ± 0.21	34.50 ± 0.74
14	36.13 ± 3.25	35.93 ± 2.34	36.02 ± 1.21	36.02 ± 0.19	35.50 ± 0.12	35.93 ± 1.80

The weight of each mouse was determined daily during the all experiment; The data are average values for 6 animals/group; The letters show significant statistical differences as follows: ^a^ with respect to the control value, and ^b^ with respect to value in group treated with B[a]P. Analysis of variance and Tukey-Kramer tests (a ≤ 0.05).

**Table 2:** Food consumption (g) in mice treated with cranberry ethanolic extract (CEE) and benzo[a]pyrene (X ± SD).

Day	Control	CEE 800 mg/kg	B[a]P 200 mg/kg	CEE + B[a]P 200 + 200 mg/kg	CEE + B[a]P 400 + 200 mg/kg	CEE + B[a]P 800 + 200 mg/kg
Treatment period						
0	2.91 ± 0.08	3.00 ± 0.0	2.82 ± 0.08	2.97 ± 0.12	3.07 ± 0.0	3.03 ± 0.01
1	2.98 ± 0.12	3.00 ± 0.07	3.05 ± 0.26	3.08 ± 0.36	3.10 ± 0.02	3.09 ± 0.08
2	3.03 ± 0.09	3.03 ± 0.04	3.06 ± 0.22	3.16 ± 0.09	3.17 ± 0.07	3.13 ± 0.14
3	3.13 ± 0.12	3.10 ± 0.07	3.19 ± 0.26	3.17 ± 0.11	3.19 ± 0.01	3.18 ± 0.17
4	3.25 ± 0.12	3.16 ± 0.07	3.20 ± 0.26	3.22 ± 0.11	3.19 ± 0.01	3.20 ± 0.26
5	3.35 ± 0.12	3.33 ± 0.07	3.30 ± 0.26	3.32 ± 0.11	3.37 ± 0.01	3.35 ± 0.17
6	3.50 ± 0.09	3.48 ± 0.26	3.69 ± 0.22	3.66 ± 0.09	3.44 ± 0.02	3.47 ± 0.04
7	3.57 ± 0.23	3.60 ± 0.08	3.82 ± 0.41	3.79 ± 0.05	3.52 ± 0.02	3.55 ± 0.04
8	4.02 ± 0.30	3.95 ± 0.26 ^b^	4.12 ± 0.22 ª	4.12 ± 0.10 ª	3.91 ± 0.01^b^	3.90 ± 0.07 ^b^
Recovery period						
9	4.07 ± 0.30	3.98 ± 0.26	4.12 ± 0.26	4.06 ± 0.30	3.99 ± 0.26	4.00 ± 0.17
10	4.08 ± 0.10	4.02 ± 0.04	4.10 ± 0.36	4.00 ± 0.14	4.03 ± 0.22	4.05 ± 0.05
11	4.06 ± 0.23	4.04 ± 0.23	4.09 ± 0.24	3.99 ± 0.17	4.04 ± 0.11	4.02 ± 0.07
12	4.00 ± 0.07	4.06 ± 0.01	4.05 ± 0.14	4.01 ± 0.04	4.02 ± 0.07	3.99 ± 0.12
13	3.99 ± 0.11	3.97 ± 0.10	3.98 ± 0.07	4.03 ± 0.07	3.98 ± 0.04	4.03 ± 0.01
14	4.02 ± 0.09	4.08 ± 0.02	4.00 ± 0.02	4.03 ± 0.0	4.00 ± 0.01	4.06 ± 0.02

The amount of food ingested was determined daily per cage by obtaining the difference in weight before and after each measurement; The data are average values for 6 animals/group; The letters show significant statistical differences as follows: ^a^ with respect to the control value, and ^b^ with respect to value in group treated with B[a]P. Analysis of variance and Tukey-Kramer tests (a ≤ 0.05).

**Table 3:** Frequency of normochromatic micronucleated erythrocytes in mice treated with CEE and benzo[a]pyrene (X ± SD).

Day/hours	Control	CEE 800 mg/kg	B[a]P 200 mg/kg	CEE + B[a]P 200 + 200 mg/kg	CEE + B[a]P 400 + 200 mg/kg	CEE + B[a]P 800 + 200 mg/kg
Treatment period						
0/0	0.75 ± 0.34	0.66 ± 0.16	0.66 ± 0.21	0.83 ± 0.16	0.66 ± 0.21	0.66 ± 0.21
2/48	1.00 ± 0.21	0.16 ± 0.21	1.50 ± 0.56	1.33 ± 0.21	1.50 ± 0.21	1.33 ± 0.22
4/96	0.50 ± 0.21	0.16 ± 0.16	3.60 ± 0.42 ^a^	3.50 ± 0.22	3.00 ± 0.10	2.33 ± 0.16 ^b^
6/144	0.75 ± 0.34	0.50 ± 0.16	4.83 ± 0.30 ^a^	3.33 ± 0.22 ^b^	3.33 ± 0.16 ^b^	2.50 ± 0.25 ^b^
8/192	1.00 ± 0.36	1.00 ± 0.16	4.83 ± 0.33 ^a^	4.33 ± 0.22 ^b^	2.50 ± 0.21 ^b^	1.65 ± 0.33 ^b^
Recovery period						
10/240	0.16 ± 0.40	0.16 ± 0.22	2.50 ± 0.21 ^a^	1.33 ± 0.42 ^b^	0.86 ± 0.56 ^b^	1.33 ± 0.16 ^b^
12/288	1.00 ± 0.21	1.00 ± 0.16	1.50 ± 0.42	0.16 ± 0.22	0.66 ± 0.21	0.66 ± 0.34
14/336	1.00 ± 0.34	0.83 ± 0.30	1.00 ± 0.56	0.50 ± 0.22	0.16 ± 0.16	0.50 ± 0.25

One thousand erythrocytes per animal stained with Giemsa were scored to determine the rate of micronucleated normochromatic erythrocyte rate; Values represent the mean ± S.D. of six mice per group; The letters show significant statistical differences as follows: ^a^ with respect to the control value, and ^b^ with respect to value in group treated with B[a]P. Analysis of variance and Tukey-Kramer tests (a ≤ 0.05).

**Table 4:** Relationship between the number of polycromatic erythrocytes with respect to the number of normochromatic erythrocytes (PE/NE index).

Day/hours	Control	CEE 800 mg/kg	B[a]P 200 mg/kg	CEE + B[a]P 200 + 200 mg/kg	CEE + B[a]P 400 + 200 mg/kg	CEE + B[a]P 800 + 200 mg/kg
Treatment period						
0/0	0.010 ± 0.001	0.012 ± 0.001	0.010 ± 0.001	0.012 ± 0.001	0.011 ± 0.001	0.009 ± 0.0009
2/48	0.008 ± 0.0008	0.011 ± 0.001	0.006 ± 0.008	0.008 ± 0.0009	0.008 ± 0.001	0.009 ± 0.0003
4/96	0.010 ± 0.0004	0.013 ± 0.002	0.003 ± 0.008 ^a^	0.005 ± 0.001	0.006 ± 0.0009 ^b^	0.008 ± 0.0009 ^b^
6/144	0.010 ± 0.001	0.013 ± 0.001	0.003 ± 0.0007 ^a^	0.005 ± 0.0005	0.004 ± 0.001 ^b^	0.008 ± 0.0002 ^b^
8/192	0.010 ± 0.0007	0.010 ± 0.0009	0.003 ± 0.0007 ^a^	0.004 ± 0.0006 ^b^	0.004 ± 0.001 ^b^	0.008 ± 0.001 ^b^
Recovery period						
10/240	0.011 ± 0.001	0.014 ± 0.0025	0.007 ± 0.001 ^a^	0.009 ± 0.001	0.010 ± 0.001 ^b^	0.009 ± 0.0009
12/288	0.010 ± 0.007	0.011 ± 0.009	0.011 ± 0.007	0.010 ± 0.006	0.011 ± 0.001	0.008 ± 0.001
14/336	0.011 ± 0.001	0.013 ± 0.001	0.011 ± 0.001	0.010 ± 0.001	0.012 ± 0.005	0.009 ± 0.0004

Mice treated with cranberry ethanolic extract (CEE) and benzo[a]pyrene (X ±SD); The amount of each type of erythrocyte was determined in 1,000 erythrocytes per animal using the Giemsa stain; Values represent the mean ± S.D. of six mice per group; The letters show significant statistical differences as follows: ^a^ with respect to the control value, and ^b^ with respect to value in group treated with B[a]P. Analysis of variance and Tukey-Kramer tests (a ≤ 0.05).

## 3. Discussion

In the last years, diverse types of berry fruits have been gaining the attention of different researchers as functional foods due to their well documented protection against urinary tract infections and inhibition of cell proliferation. This last property has been related, at least in part, to a multitude of bioactive phytochemicals that these colorful fruits contain, including flavonoids (anthocyanins, flavonols, and flavanols), tannins (proanthocyanidins, ellagitannins, and gallotannins), quercetin, phenolic acids, lignans, and stilbenoids. Different studies and researchers agree that the anticancer effects of berry bioactives are partially mediated through their abilities to counteract, reduce, and also repair damage resulting from oxidative stress and inflammation [3,5,10,15,26,27,28]. Based on this background, we evaluated the ability of a cranberry ethanolic extract to counteract the toxic effects of benzo[a]pyrene, a compound belonging to the group of the polycyclic aromatic hydrocarbons (PAH). This polycyclic aromatic hydrocarbon has been widely analyzed, and studied for many years due to its high toxicity. Several authors using different types of assays have indicated his mutagenic, carcinogenic, teratogenic and clastogenic capacity [29,30,31,32]. 

However, with respect to the effect produced by this PAH on the body weight there are contradictory results. Some studies have indicated that when it is administered orally, by inhalation, or through intraperitoneal injection in doses ranging from 50 to 2,250 mg/kg the body weight might diminish in experimental animals [33,34,35,36,37]. There is also information that show the effects of smoking a large amount of tobacco, which is one of the main sources of contamination with benzo[a]pyrene (20–50 ng of B[a]P per cigarette), that might also reduce the body weight [38,39]. Likewise, other studies performed by Knuckels *et al*. where was quantified the body weight and analized the histopathology of some organs (liver, kidney, stomach, prostate, testicles and ovaries) during 30, 60 and 90 days after administration of benzo[a]pyrene in three doses (5, 50, and 100 mg/kg) by oral gavage to rats F-344 (females and males) showed a significant reduction in the body weight with the highest dose (100 mg/kg). Their results indicated that the body weight can diminishes and concluded that this effect is more in males than in females [40]. In this sense, our study demonstrated the opposite, for a trend to increase the body weight is shown during the last days of treatment (day 7 and 8) and the first days of the recovery period (days 9 and 10). This kinetics was similar to the study performed by Irigaray in which C57BI/6J mice were chronically intoxicated with a 0.5 mg/kg of B[a]P dose by intraperitoneal injection for 15 days. An increase of 43% in the weight was observed in comparison to the control group. It was also found that when the polycyclic aromatic hydrocarbon was removed, the excess in weight increasing was not immediately corrected, which was an effect similar to ours experiment [41]. The same researchers showed that even though there was no change in food intake, the animals gained weight. This result was different from ours only in day 8 of treatment, for during the recovery week this parameter was not affected.

Although the mechanism or mechanisms responsible for the control of the body weight are not completely known, a possible explanation could be that the B[a]P caused a lipolysis inhibition at blocking the ACTH receptors and β-adrenergic receptors (specifically, agonists β1, β2 and β3) which signals are coupled with the Protein G system [42,43]. In regards to the weight increase and the reason why this parameter was not immediately corrected at the PAH elimination, we might suggest some possibilities: the first, would be that a subchronic or chronic exposure to B[a]P induces to significant changes in the adipose tissue metabolism; the second possibility may be related to the formation of chemical interactions between the mutagen and the adipose cell (maybe due to its high lipophilicity), and finally, that the B[a]P could have a long half-life due to the subchronic or chronic exposure and the deposit in the same tissue, which requires a longer time to revert the effect on body weight. The previous possibilities suggest that the weight increase is due to an increase in the body mass index (BMI), provoked by a fat accumulation when the lipolysis was inhibited and, at the same time, this lipid accumulation caused a delay in the B[a]P elimination. With respect to the reason why the food ingestion was not modified, there are evidences that in “β-less” mice in which none of the three beta-adrenergic receptors are activated, an increase in the body weight is perceived without any change in the food ingestion [44]. Besides, it has been observed that some medications used by humans that block the same receptors diminish the energy consumption and increase the fat mass without alteration in the food ingestion [45]. 

The present study also showed that cranberry ethanolic extract (CEE) did not affect the body weight or the food ingestion or was a genotoxic or cytotoxic agent either, but reduced significantly the toxicity and genotoxicity produced of benzo[a]pyrene. It is important to mention that in the literature there are only a few *in vivo* studies where the same indicators or the same species of cranberries are analyzed (*Vaccinium macrocarpon)*. Investigations performed by Barros *et al*. demonstrated that the ingestion of berries is beneficial in reversing neuronal changes related to aging and that it does not affect the body weight in adult male Swiss mice when they are treated with an extract of *Vaccinium ashei* for 30 days [23]. Although this extract belongs to a different species from ours and that the nutritional composition may vary, the effect on the body weight was the same; thus, we may suggest that the *Vaccinium* gender possibly does not modify this parameter of evaluation. Some evidences have indicated that the cranberries juice might induce cytochrome enzymes P450 [46,47], which possibly favors the elimination of B[a]P and prevents the alteration in the body weight or the food ingestion and thus, protects the organism.

In order to analyze the antigenotoxic and anticytotoxic capability of CEE, it should be remembered that this polycyclic aromatic hydrocarbon is rapidly adsorbed by the intestine and, due to its highly lipophilic nature it is transported in plasma through the lipoprotein system [48,49]. Studies on the distribution of B[a]P in tissues have demonstrated their accumulation in storage lipids, including the mammary glands and the adipose tissue. This contaminant is metabolized through the cytochrome system P450 (specifically CYP1A1, CYP1A2, CYP1B1) in reagents derived from dihydrodiol epoxide (e.g., B [a] P-7,8-dihydrodiol-10-epoxide or BPDE). These metabolites join DNA covalently which results in adducts that lead to mutations, uncontrolled growing of cells and therefore, to the formation of tumors in different tissues (lung adenocarcinoma, lymphoproliferative tumors, hepatomas, mammary adenocarcinomas) [41]. Besides, at the constant exposure to this substance an oxidative damage to the DNA is induced which plays an important role in the carcinogenic process due to the fact that the B[a]P produces quinone derivatives that easily generate reactive oxygen species (ROS) which are involved in the formation of chromosome abnormalities, DNA strand breaks and to its clastogenic capacity [50]. The dose used in this experiment (200 mg/kg), confirm again the genotoxic capacity of the benzo[a]pyrene and, together with the experiments performed by other authors agree that this mutagen increases the frequency of micronuclei in reticulocytes and erythrocytes (polychromatic and/or normochromatic), when it is administered in doses with ranges between 125 and 250 mg/kg [51,52,53,54]. On the other hand, we confirm that cranberry ethanolic extract (CEE) showed a dose-dependent protection; the most significant antigenotoxic effect was with 800 mg/kg. In this aspect, it is important to recall that there is no precedent where the antigenotoxic capacity has been assessed with the micronuclei technique *in vivo* and in a subchronic form. The few studies that exist concerned acute exposures and analyzed another *Vaccinium* species. Research with strawberry and cherry sweets concentrates (administered orally) both belonging to the berry-fruit group and directly related to the *Vaccinium* gender reduced in a moderately (approximately 40%) way the amount of micronucleated polychromatic erythrocytes (MNPCE) induced with the same mutagen and a quite similar dose to the one we used (150 mg/kg) [52]. The results were compared afterwards with the protector effect of quercetin (a phytochemical present in the chemical composition of *Vaccinium macrocarpon,* which the species that CEE comes from) and its glycoside, isoquercitrin. Both compounds reduced the number of micronuclei in polychromatic erytrocytes of the bone marrow of mice in approximately 73 and 33% respectively [54]. Furthermore, researches with an extract of *Vaccinium ashei* administered during 30 days orally diminishes the DNA damage induced by H_2_O_2_ in the brain tissue (hippocampi and cerebral cortices) evaluated with alkaline single cell electrophoresis (comet) assay [23].

Therefore, at comparing the results of our experiment with the investigations mentioned we can consider that: (a) the micronuclei technique was the suitable technique to evaluate the genotoxic potential of BaP, as well as, the protective capacity of the cranberry ethanolic extract (CEE); and (b) the extract belongs to the *Vaccinium macrocarpon* species and according to the evidences shown as well as in the conditions of this study, we can suggest that it is not a toxic agent, for it does not modify the body weight, the food ingestion, or the genetic material and, finally; (c) the antigenotoxic capacity of cranberry ethanolic extract can thus be attributed to the individual or combined effect of some phytochemicals constituents of the fruit, such as flavonoids (anthocyanins), tannins (proanthocyanidins), and quercetin, which are known to have anticancer properties [3,11,16,17,18,25,55]. Our findings are of importance as plant or fruits extract can be used as a natural dietary supplement to counteract the cytotoxic effects resulting from exposure to the mutagens and carcinogens exposed through drugs, diet or environment. The chemopreventive action of plants or fruits extracts have been related to their ability to enhance the activities of carcinogen metabolizing enzymes and/or to bind with toxicants thus reducing their effective critical concentrations. Alternatively, they also act as antioxidants and counteract the increased amount of oxidants generated by the toxicants (such as, B [a] P-7, 8-dihidrodiol- and BPDE produced by the benzo[a]pyrene). Influence of fruits products, for example, flavonoids on the activity of cytochrome P450-dependent enzymes has been reported and it is suggested that it contributes to the anticancer activity of flavonoids [56]. Quercetin is another substance has shown demostrated the same properties [25]. Thus, at present it is suggested that extract exerts its protective effects by modulating the activities of cytochrome P450 enzymes; besides, its antioxidant properties may also contribute to overall protective effects as observed in our studies.

## 4. Experimental

### 4.1. Chemicals

The following compounds were purchased from Sigma Chemicals (St. Louis, MO, USA): benzo[a]pyrene (B[a]P), methanol, ethanol, monobasic potassium phosphate (KH_2_PO_4_) and sodium phosphate dibasic (Na_2_HPO_4_). The Giemsa stain was obtained from Merck (Mexico City).

### 4.2. Animals

All the animals were male mice strain CD-1 with a mean weight of 25 g. They were obtained from the National School of Biological Sciences (Mexico City), and maintained in our pharmacology laboratory in metallic cages at a temperature of 23 ± 2 °C and 50 ± 10% humidity, with food (TEKLAD Global 2018S, Harlan, Mexico City) and water *ad libitum*, in a 12 h light-dark period. The adjustment time period for the animals was one week before the treatments.

### 4.3. Experimental Determinations

#### 4.3.1. Preparation of Cranberry Ethanolic Extract

The cranberries used in the experiment were obtained at the market of Zacatlan, a town located in the State of Puebla, Mexico. Once the fruits were purchased, we cleaned them with tap water and selected the less damaged pieces. The fruit weighed 300 g (approximately, 600 cranberries). They were put in a previously weighed round bottomed flask and ethanol (300 mL) was added. The mixture was left in the fridge for 3 days. After this time, 6 mL (1 N) of hydrochloric acid was added and allowed to stand for 1 h. Subsequently, the flask was introduced in an ultrasonic bath with movements of 5 cycles for 1 min with breaks of 2 min between each cycle. At the end of the time, the pulp was filtered and separated from the liquid. Finally, the ethanol was evaporated on a rotary evaporator (Büchi, Flawil, Switzerland) at a temperature of 45 °C with a pressure of 50 mmHg to give the cranberry concentrate. Approximately, 37.69 g of cranberry ethanolic extract (CEE) were obtained according to the weight difference of the flask. The extract was maintained at 4 °C in the dark until it was used.

#### 4.3.2. LD50 of Cranberry Ethanolic Extract

A procedure that required few animals was followed for this determination [57]. The assay was made in two steps: a single oral administration of CEE in doses of 10, 100 and 1,000 mg/kg body weight (bw) were tested first, and showed no lethality among the animals; then the effect of 1,600, 2,900, 4,300 and 5,600 mg/kg bw was tested, and no mortality appeared among the treated mice either. With the result of this experiment, we considered to use doses of 200, 400 and 800 mg/kg bw for the determination of the genotoxic and antigenotoxic potential.

#### 4.3.3. Genotoxicity/Antigenotoxicity Protocols

The protocol was approved by the Committee of Ethics and Biosecurity of the Institute of Health Sciences. The animals were organized in groups of six individuals each, as follows: a negative control group (without any treatment), a positive group treated with benzo[a]pyrene (200 mg/kg body weight, dissolved in 200 μL corn oil), a group administered with 800 mg/kg of cranberry ethanolic extract (CEE), and three groups treated with B[a]P and cranberry ethanolic extract (200, 400, and 800 mg/kg bw) respectively. The cranberry ethanolic extract and B[a]P were administered orally by intragastric gavage for a week on a daily basis according to the experimental groups mentioned. During this period the body weight, the feed intake and the determination of genotoxic and/or antigenotoxic potential of the compounds were quantified. At the end of this period, the chemicals were removed, and the determinations continued for one week more (recovery period).

Micronucleus assay was used to determine the genotoxic and antigenotoxic capacity of the compounds. The rate of micronucleated normochromatic erythrocytes (MNNE) was determined before the experimental treatment, and at 48, 96, 144, 192, 240 and 336 hours after the treatment. Two blood smears were made from the tail of each animal, fixed in methanol for 5 min and stained for 20 min with a 4% Giemsa solution made in phosphate-buffered saline, at a pH of 6.8 [58]. One thousand erythrocytes per animal were scored to determine the rate of MNNE. In order to evaluate the bone marrow cytotoxicity, we scored 1,000 erythrocytes per animal and established the rate of polychromatic erythrocytes (PE) with respect to the number of normochromatic erythrocytes (PE/NE index). 

The weight of each mouse was determined daily during the 14 days of the study (treatment and recovery period). We provided the same amount of food in each cage and measured the weight of food daily. The difference in weight per cage indicated the food consumed. All the data obtained were analyzed with the statistical program INSTAT version 3.0, ANOVA and were performed to determine if the data among all groups were normally distributed, and in that case Tukey-Kramer tests as a post-test were performed to establish the statistical differences between groups in each assay.

## 5. Conclusions

In the present investigation we have demonstrated that treatment with cranberry ethanolic extract (CEE) significantly prevents the damage induced by benzo[a]pyrene in mice, thereby reducing the frequency of micronuclei. Our data also suggest that the antioxidant capacity of the extract may be involved in that effect.
